# Draft Genome Sequences of Three Novel Acinetobacter Isolates from an Irish Commercial Pig Farm

**DOI:** 10.1128/MRA.00919-19

**Published:** 2019-09-26

**Authors:** Ana Pereira do Vale, João Anes, Séamus Fanning, Finola Leonard, Damien Farrell

**Affiliations:** a UCD School of Veterinary Medicine, University College Dublin, Dublin, Ireland; b Institute of Technology Sligo, School of Science, Sligo, Ireland; c UCD Centre for Food Safety, University College Dublin, Dublin, Ireland; University of Arizona

## Abstract

Acinetobacter species are important in the emergence and spread of antimicrobial resistance (AMR), which threatens human and animal health worldwide. Here, we present the draft genome sequences of three Acinetobacter species strains (RF14B, RF15A, and RF15B) isolated from pig feces and the floor of a pig hospital pen in Ireland.

## ANNOUNCEMENT

Some Acinetobacter species, including Acinetobacter baumannii, are multidrug-resistant bacteria responsible for infections in hospital settings. Intensive animal production sites are similar to hospitals in many respects, and the role of Acinetobacter in the microbiota of intensively farmed pigs needs to be investigated with care to evaluate their potential to spread antimicrobial resistance (AMR) genes (1). In this study, we present the draft genome sequences of three strains from an Irish farrow-to-finish commercial pig farm.

RF14B was isolated from pig feces, and strains RF15A and RF15B were isolated from the floor of a hospital pen. Environmental swabs were obtained as described by Mannion et al. (2). After incubation at 37°C for 20 hours, individual colonies were selected. Isolates were grown in Luria-Bertani broth overnight. Genomic DNA was extracted using the UltraClean microbial DNA isolation kit (Mo Bio Laboratories, Carlsbad, CA) according to the manufacturer’s instructions. Genomic libraries were prepared using the NEBNext Ultra II fragmentation system (FS) (New England Biolabs, Dublin, Ireland), according to the manufacturer’s recommendations, and sequenced on the MiSeq platform (Illumina, San Diego, CA) using 2 × 300-bp paired-end reads.

The reads were *de novo* assembled with SPAdes v3.10.0 (3) using default settings. The quality of the subsequent assemblies was assessed using QUAST (4). For the purpose of building a phylogenetic tree, assemblies of isolates and some reference species were annotated using Prokka v1.12 (5), and the core genome was calculated using Roary (6) with an identity threshold of 90%. Final annotation was done using the NCBI Prokaryotic Genome Annotation Pipeline (PGAP) (7) during genome submission. Average nucleotide identity (ANI) values were calculated using the Pyani package (8). Screening for multiple resistance and virulence genes was run on the assembled contigs using ABRicate v0.8.3 (9) with the following databases for each category of gene: antimicrobial resistance genes were identified using the Comprehensive Antibiotic Resistance Database (CARD) (10), contigs with plasmid replicons were determined using PlasmidFinder (11), and virulence factors were detected using the Virulence Factors Database (VFDB) (12). A phylogenetic tree was constructed using the R packages Phangorn v2.5.5 (13) and APE v5.3 (14). The tree was built using an alignment of the core genome sequences derived from Roary with the maximum likelihood method and bootstrapped 100 times. Default parameters were used for all software unless otherwise specified. Assembly details are shown in Table 1.

**TABLE 1:** Assembly and annotation metrics

Strain	Avg coverage (×)	No. of contigs	*N*_50_ (kb)	Assembly length (kb)	G+C content (%)	No. of CDSs^*a*^
RF14B	57	257	25,955	2,926,145	43.41	2,788
RF15A	60	66	133,703	3,022,426	43.55	2,695
RF15B	56	57	167,102	3,028,007	43.55	2,701

a CDSs, protein-coding sequences.

**FIG 1:**
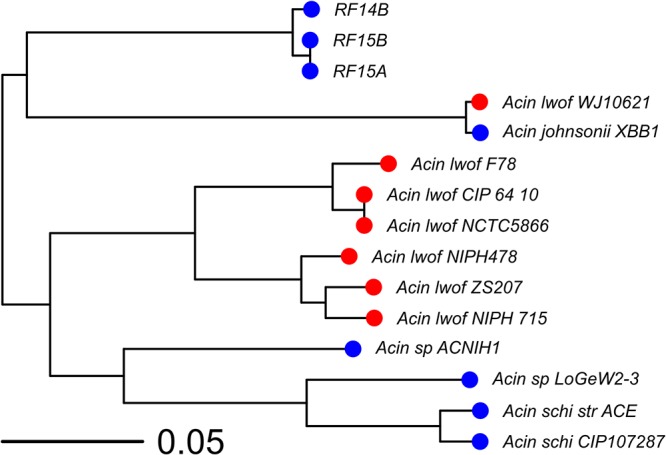
Midpoint rooted phylogenetic tree reconstructed with the maximum likelihood method using the core genome alignment between our isolates and multiple related Acinetobacter species. Acinetobacter lwoffii strains are colored with red circles. The bootstrap support was 100% at each node.

The two hospital pen isolates had >99.9% average nucleotide identity (ANI) to each other and 98% identity to the fecal isolate. The closest Acinetobacter species is strain ACNIH1 with an ANI of 85%. The phylogenetic tree in Fig. 1 shows the relationship of these isolates to the closest known species. Genes *aadA1*, *dfrA1*, and *sat-1*, usually associated with mobile genetic elements, were found in both hospital pen isolates. Extended-spectrum β-lactamase (ESBL) CTX-M-144 was detected in RF15B with partial coverage of 53.39%. Additionally, *adeI*, *adeJ*, and *adeK* (adeABC pumps), associated with carbapenem resistance when overexpressed, were identified in all three isolates.

Further comparative genomic analyses with closely related Acinetobacter species will provide insights into the sequence novelty of these strains and their true phylogenetic status.

### Data availability.

The draft genome assemblies of the three isolates have been deposited at DDBJ/ENA/GenBank under the accession numbers SMTB00000000, VLSQ00000000, and VLSR00000000. The versions described here are the first versions. The BioProject number is PRJNA427141, and the BioSample numbers are SAMN08224478, SAMN08224479, and SAMN08224480. The raw sequencing data are available in the Sequence Read Archive (SRA) under the accession numbers SRR6409923, SRR6409922, and SRR6409912.
